# Protein Synthesis Errors and Longevity: A Lesson from a Single Amino Acid Mutation Study

**DOI:** 10.14336/AD.2021.1211

**Published:** 2022-02-01

**Authors:** Shan Liang, Dong-Yi Li, Jun-Hao Wen, Ji-Xin Tang, Hua-Feng Liu

**Affiliations:** Key Laboratory of Prevention and Management of Chronic Kidney Disease of Zhanjiang, Institute of Nephrology, Affiliated Hospital of Guangdong Medical University, Zhanjiang 524001, Guangdong, China.

**Dear Editor,**

**Figure 1.**
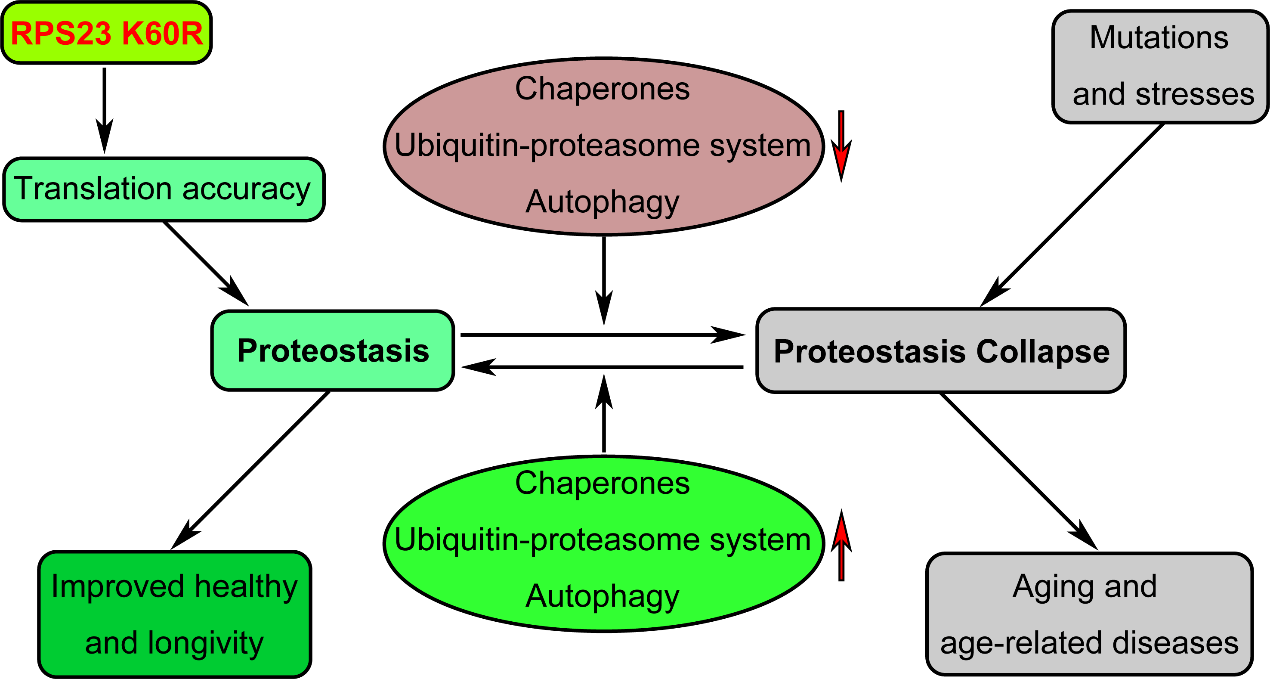
A single amino acid mutation in the decoding center of the ribosome, RPS 23 K60R, was able to increase protein synthesis fidelity and improve health and longevity of organisms. Proteostasis is critically involved in healthy aging and longevity. Chaperones, ubiquitin-proteasome systems and autophagy are the main players in proteostasis maintenance, and most of them have been proved to play an essential role in health and longevity of organisms. Bjedov, Cabreiro and colleagues work showed another way to improve health and longevity of organisms through a single amino acid mutation in the ribosomal decoding center, RPS 23 K60R, which could increase fidelity of protein synthesis and extend lifespan of yeasts, worms, and flies.

Proteostasis refers to the ability that cells maintain the balance among protein synthesi, folding and degradation, which is a formidable task, considering that 10,000 to 20,000 different proteins exist in a mammalian cell and these proteins are only marginally stable in physiological state (Fig. 1). Proteostasis collapse, the failure to keep proteostasis, has been established as a hallmark of aging and age-related diseases. Giving that protein synthesis is one of the key determinants to maintain proteostasis, therefore, increasing the protein synthesis fidelity may involve in the organismal health and longevity. Writing in Cell metabolism, Bjedov, Cabreiro and colleagues recently reported that a single amino acid mutation in the ribosomal decoding center is able to increase the protein synthesis fidelity and extend lifespan of yeasts, worms, and flies [1](Fig. 1).

Previous study had demonstrated that a strong correlation between the translation fidelity and the maximum lifespan in 16 rodent species, and translation fidelity, which coevolved with longevity, was regarded as a key factor in determining rodent species lifespan [2]. In yeasts, the translation fidelity had also been found to be a determinant of health and longevity [3, 4]. However, although a lot of works have been done in single cell organisms and in vitro cultured cells [5], the physiology role of translation fidelity in the multi-cellular organisms are rarely investigated, and the effect of increasing protein synthesis accuracy in health and longevity in metazoan organisms remains unexplored.

Bjedov, Cabreiro and colleagues therefore focused on exploring the way to increase protein synthesis accuracy in multi-cellular organisms and examined the effect of increased protein synthesis fidelity on health and longevity in metazoan species [1].

First, to identify the key determinant to increase protein synthesis accuracy, the authors used different databases to perform phylogenetic analysis of RPS23, which had been found to be mutated in the most well-described hyperaccuracy *E. coli* mutants [6, 7], in organisms ranging from archaea to eukaryotes. They found a conserved lysine residue in the KQPNSA region of ribosomal RPS23, which was nearly invariant throughout evolution but only changed in some thermophilic and hyper-thermophilic archaea, where the amino acid lysine was substituted by arginine.

Next, the authors investigated the function of lysine (K)-to-arginine (R) substitution in translation accuracy by introducing a K60R mutation to the KQPNSA region of *Drosophila*, *Caenorhabditis elegans* and *Schizos-accharomyces pombe* rps23 using CRISPR/Cas9 or standard genetic techniques. They found that a single amino acid substitution, RPS23 K60R, in the ribosomal decoding center was able to increase protein synthesis accuracy in *Drosophila*, *Caenorhabditis elegans* and *Schizosaccharomyces pombe*.

In addition, the authors also investigated the physiological function of RPS23 K60R mutation in the mutant organisms through measuring heat stress resistance and development speed. They found that these RPS23 K60R mutant yeasts, worms, and flies had a greater capacity to resist heat stress, but had an obvious developmental delay.

Furthermore, the authors further investigated whether the increased protein synthesis fidelity can extend lifespan in both single and multi-cellular organisms. Remarkably, they found that these RPS23 K60R mutant yeasts, worms, and flies had a significant lifespan extension, with 9%-23% lifespan extension in the all repeated assays. Moreover, the authors also investigated if these long-lived mutant organisms were much healthier than the normal controls. Using a negative geotaxis or climbing assay, they found that RPS23 K60R mutant flies had an improved climbing ability compared to controls, suggesting that the mutants were much healthier.

Finally, the authors also explored whether the well-studied anti-aging drugs, such as rapamycin, Torin1, and trametinib, can improve translation fidelity. They found that all these three anti-aging drugs were able to increase protein synthesis fidelity, suggesting that translation fidelity may be the common target of the well-known pharmacological anti-aging interventions. Moreover, they also investigated if these pharmacological anti-aging interventions can further extend the lifespan of RPS23 K60R mutants. They treated the RPS23 K60R mutant yeasts, worms, and flies with rapamycin and found that rapamycin was able to extend further the longevity of these mutants, implying that translation fidelity indeed a common target of the diverse pharmacological anti-aging therapies.

In summary, these findings confirmed the essential role of translation accuracy in maintaining proteostasis, improving health and longevity of metazoan species, and established the broad therapeutic potential of targeting translation fidelity for aging and age-related diseases. Moreover, this work also showed a way to increase protein synthesis fidelity to extend lifespan in multi-cellular organisms. Remarkably, a single amino acid mutation in the decoding center of the ribosome has such a big effect on improving health and longevity in yeasts, worms, and flies. Further studies are needed to determine whether mutating a single amino acid in the same site is able to increase protein synthesis fidelity and improve health and longevity in mammals, and to elucidate the specific mechanism.
